# Intrapartum Fetal Heart Rate: A Possible Predictor of Neonatal Acidemia and APGAR Score

**DOI:** 10.3389/fphys.2018.01489

**Published:** 2018-10-22

**Authors:** Thâmila Kamila de Souza Medeiros, Mirela Dobre, Daniela Monteiro Baptista da Silva, Andrei Brateanu, Ovidiu Constantin Baltatu, Luciana Aparecida Campos

**Affiliations:** ^1^Center of Innovation, Technology and Education at Anhembi Morumbi University – Laureate International Universities, São José dos Campos, Brazil; ^2^School of Health Sciences at Anhembi Morumbi University – Laureate International Universities, São José dos Campos, Brazil; ^3^Division of Nephrology and Hypertension, University Hospitals, Cleveland, OH, United States; ^4^Medicine Institute, Cleveland Clinic, Cleveland, OH, United States

**Keywords:** fetal monitoring, heart rate, perinatal outcome, noninvasive prenatal diagnosis, neonatal acidemia, APGAR

## Abstract

**Background:** Predicting perinatal outcomes based on patterns of fetal heart rate (FHR) remains a challenge. The aim of this study was to evaluate intrapartum FHR variability as predictor for neonatal acidemia and APGAR score.

**Methods:** This was a retrospective observational study of 552 childbirths. Multivariable linear regression models were used to assess the association between FHR variability and each of the following outcomes: arterial cord blood pH and base deficit, Apgar 1, and 5 scores. Variables used for adjustment were maternal age, comorbidities (gestational diabetes, preeclampsia, maternal fever, and hypertension), parity, gravidity, uterine contractions, and newborn gestational age, and weight at birth.

**Results:** The following factors were associated with an increased risk of metabolic acidosis and low Apgar scores at birth: increased mean and coefficient of variation (CV) of the FHR, type of delivery and decreased parity. Each 10-beat/min increase in the FHR was associated with an increase of 0.43 mEq/L in the base deficit, and a decrease of 0.01 in the pH, 0.2 in the Apgar 1, and 0.14 in the Apgar 5 scores. Each 10% increase in the CV of the FHR was associated with an increase of 4.05 mEq/L in the base deficit and a decrease of 0.13 in the pH, 1.31 in the Apgar 1, and 0.86 in the Apgar 5 scores.

**Conclusion:** These data suggest the intrapartum FHR variability is physiologically relevant and can be used for predicting the acidemia and Apgar scores at birth of the newborn infants without severe cases of morbidity and from uncomplicated pregnancies.

## Introduction

Contemporary research aims to identify reliable and early markers for the neonatal acidemia and the physical condition of a newborn infant (Devane et al., 2012, 2017). Intrapartum cardiotocography monitors fetal heart rate (FHR) and uterine contractions and is commonly used for the early detection of fetal distress (Stout and Cahill, 2011).

It is theorized that intrapartum cardiotocography FHR could detect fetal hypoxia and/or acidosis allowing a timely intervention to reduce adverse neonatal outcomes such as postnatal cerebral palsy. This is based on the theory that intrapartum hypoxia may lead to alterations in the fetal central nervous system that directly affects the electrical activity of the fetal heart and could also induce neonatal cerebral palsy (Garabedian et al., 2017). Indeed, cardiotocography FHR parameters including baseline FHR and its variability appear to be independent predictors of fetal acidosis (Silberstein et al., 2017), were associated with a substantial decrease in early neonatal mortality and morbidity (Chen et al., 2011). At present, however, there is no consensus regarding sensitivity and specificity of cardiotocography classifications in predicting acidemia, with three guidelines for cardiotocography interpretation provided by the International Federation of Gynecology and Obstetrics (FIGO), American College of Obstetrics and Gynecology (ACOG), and National Institute for Health and Care Excellence (NICE) (Bhatia et al., 2017; Santo et al., 2017).

The improvement in the accuracy of FHR pattern interpretation through a continuous FHR centralization system is envisaged to be beneficial in reducing the incidence of neonatal acidemia (Michikata et al., 2016). To improve the predictive efficacy of cardiotocography FHR, diagnostic algorithms are currently being investigated to be used in real-time computerized systems for decision support (van Scheepen et al., 2016).

In this study, we aimed at investigating whether the intrapartum baseline and variability parameters of FHR are independently associated with neonatal acidemia and the APGAR scores of the newborn infants without severe cases of morbidity and from uncomplicated pregnancies.

## Materials and Methods

### Study Design and Setting

A single-center retrospective observational study of 552 childbirths at the University Hospital Brno (UHB), Czechia (Chudacek et al., 2014). The hospital records were consecutively selected to pair both clinical and cardiotocography signal parameters.

### Clinical Data

Clinical selection criteria (Chudacek et al., 2014) included maternal age (maternal age > 18 years), gravidity (only singleton, uncomplicated pregnancies), gestational week (weeks of gestation ≥ 37), delivery type (the majority of the database consists of vaginal deliveries), fetuses without known congenital defects or known intrauterine growth restriction (IUGR). Additional criteria considered were: sex of the fetus, parity, and risk factors including gestational diabetes, preeclampsia, maternal fever (>37.5°C), hypertension, and meconium stained fluid. The cohort included neonates without severe cases of neonatal morbidity, hypoxic ischemic encephalopathy, or seizures.

### Cardiotocography Data

Cardiotocography recordings included contained time information and signal of FHR and uterine contractions sampled at 4 Hz were recorded starting 90 min preceding the delivery. Cardiotocography recordings selection criteria (Chudacek et al., 2014) were the signal length: 90 min preceding the delivery with 1st stage was limited to a maximum of 60 min and 2nd stage to 30 min in order to keep recordings easily comparable. Other criteria were: missing signal, noise and artifacts, and type of measurement device. The database is composed as a mixture of recordings acquired by ultrasound doppler probe, direct scalp measurement or combination of both, reflecting the clinical reality at the obstetrics ward. Cardiotocographies were recorded using STAN S21 and S31 (Neoventa Medical, Mölndal, Sweden) and Avalon FM40 and FM50 (Philips Healthcare, Andover, MA, United States) fetal monitors.

The cardiotocography recordings and clinical data were matched using anonymous unique identifiers generated by the hospital information system. A flowchart diagram describing the process of data selection for the final database is presented in the original publication (Chudacek et al., 2014).

The acid-base status (pH and base deficit) of umbilical cord arterial blood was measured at the moment of birth to evaluate the metabolic condition of neonates.

Apgar’s scores (Apgar) at 1 and 5 min after birth evaluated the outcome of the delivery based on five categories: breathing effort, heart rate, muscle tone, reflexes, and skin color.

The CARDIOTOCOGRAPHY database is available at PhysioNet^1^ ((Chudacek et al., 2014). All Physionet databases have been fully anonymized and may be used without further institutional review board approval.

### Statistical Analysis

The FHR variability was determined using coefficient of variation (CV), which equals the standard deviation divided by the mean (Pereira et al., 2017). The characteristics of study participants were depicted using standard descriptive statistics, overall and stratified by quartiles of the mean of FHR. Chi-square (χ^2^) test of independence for categorical (nominal) variables and Analysis of variance (ANOVA) test for continuous variables were used to analyze the covariates of interest.

Multivariable linear regression models were used to assess the association between FHRVand each of the following outcomes: pH, base deficit, Apgar 1, and 5 scores. Variables used for adjustment were maternal age, comorbidities, parity, gravidity, uterine contractions, and newborn gestational age, and weight at birth.

All statistical tests were two sided, and *P* < 0.05 was considered significant. IBM Corp (2016) SPSS Statistics for MAC, Version 24.0, was used for analyses.

## Results

### Patient Characteristics

A total of 552 study participants were included in the study. The age of the mothers was 30 (27–33) median (IQR) years and gestational age was 40 (39–41) median (IQR) weeks. Characteristics of study participants distributed according to quartiles of the mean of FHR are summarized in Table 1. Patients in the lowest vs. highest quartile of the mean of FHR had a higher CV of the FHR (14.73 ± 3.56 vs. 11.24 ± 3.85). Mean of FHR was not associated with maternal age, history of diabetes mellitus or hypertension or preeclampsia, delivery type, gravidity, parity, gestational week, and mean of uterine contractions.

**Table 1:** Characteristics of study participants by quartile of mean fetal heart rate.

Characteristics		All participants	Quartiles of the mean fetal heart rate (FHR)				*P*-value
		*N* = 552 *N* (%) or Mean ± SD	*N* = 138 *N* (%) or Mean ± SD	*N* = 138 *N* (%) or Mean ± SD	*N* = 138 *N* (%) or Mean ± SD	*N* = 138 *N* (%) or Mean ± SD	
Maternal							
Age (years)		29.60 ± 4.72	30.22 ± 4.63	29.48 ± 4.47	29.15 ± 5.11	29.54 ± 4.64	0.29
History of diabetes	No	515 (93.3)	126 (91.3)	131 (94.9)	128 (92.8)	130 (94.2)	0.63
mellitus	Yes	37 (6.7)	12 (8.7)	7 (5.1)	10 (7.2)	8 (5.8)	
History of hypertension	No	508 (92)	125 (90.6)	127 (92)	127 (92)	129 (93.5)	0.85
	Yes	44 (8)	13 (9.4)	11 (8)	11 (8)	9 (6.5)	
History of Preeclampsia	No	535 (96.9)	131 (94.9)	134 (97.1)	134 (97.1)	136 (98.6)	0.38
	Yes	17 (3.1)	7 (5.1)	4 (2.9)	4 (2.9)	2 (1.4)	
Delivery type	1	506 (91.7)	127 (92)	132 (95.7)	127 (92)	120 (87)	0.07
	2	46 (8.3)	11 (8)	6 (4.3)	11 (8)	18 (13)	
Gravidity		1.41 ± 1.03	1.47 ± 1.08	1.36 ± 0.79	1.24 ± 0.79	1.55 ± 1.35	0.07
Parity		0.41 ± 0.72	038 ± 0.74	0.44 ± 0.65	0.38 ± 0.84	0.43 ± 0.66	0.83
Gestational week		39.94 ± 1.71	40.04 ± 1.11	39.81 ± 2.78	40.02 ± 1.20	39.88 ± 1.13	0.63
UC – mean (contractions/min)		24.82 ± 10.54	24.45 ± 11.48	24.16 ± 10.46	25.54 ± 10.12	25.14 ± 10.08	0.69
UC – CV		75.48 ± 25.70	74.90 ± 28.15	73.76 ± 27.29	80.70 ± 25.06	72.61 ± 21.29	0.04
Newborn							
FHR – mean (beats/min)		134,90 ± 11.73	120.85 ± 4.87	130.40 ± 2.33	138.02 ± 2.23	150.31 ± 7.19	<0.001
FHR – CV		12.86 ± 3.91	14.73 ± 3.56	12.88 ± 3.66	12.55 ± 3.77	11.24 ± 3.85	<0.001
Sex	Female	286 (51.8)	76 (55.1)	70 (50.7)	63 (45.7)	77 (55.8)	0.30
	Male	266 (48.2)	62 (44.9)	68 (49.3)	75 (54.3)	61 (44.2)	
Newborn weight (g)		3397.52 ± 459.58	3321.12 ± 442.09	3401.28 ± 472.53	3431.88 ± 447.37	3435.80 ± 471.34	0.13

*UC, uterine contractions; FHR, fetal heart rate; CV, coefficient of variation.*

### Association Between FHR and Metabolic Acidosis

The median pH values of the cohort were 7.25 (IQR: 7.17–7.3) [min to max: 6.85–7.47]. In multivariable adjusted models, the following factors were associated with an increased risk of metabolic acidosis at birth: increased mean and CV of the FHR, type of delivery (Caesarian section over vaginal), decreased parity (Tables 2, 3). The following parameters were not associated with risk of metabolic acidosis at birth: maternal age, history of diabetes mellitus, hypertension, preeclampsia, gravidity, gestational week, uterine contractions, newborn sex or weight.

**Table 2:** Association between maternal and newborn parameters and pH.

pH				
Parameters	Unstandardized B Coefficients	95% Confidence interval		*P*-value
		Lower bound	Upper bound	
Maternal age	0.000	−0.003	0.002	0.70
History of diabetes mellitus	−0.009	−0.049	0.030	0.64
History of hypertension	−0.033	−0.069	0.004	0.08
History of preeclampsia	−0.005	−0.059	0.049	0.85
Delivery type	−0.042	−0.072	−0.013	0.005
Gravidity	−0.003	−0.015	0.010	0.67
Parity	0.036	0.019	0.053	<0.001
Gestational week	0.006	−0.004	0.015	0.25
UC – mean (contractions/min)	−5.328E − 5	−0.001	0.001	0.91
UC – CV	0.000	−0.001	0.000	0.32
FHR – mean (beats/min)	−0.001	−0.002	−0.001	0.001
FHR – CV	−0.013	−0.015	−0.010	<0.001
Newborn sex	−0.013	−0.033	0.007	0.19
Newborn weight (g)	−1.108E − 5	0.000	0.000	0.34

**Table 3:** Association between maternal and newborn parameters and base deficit.

Base deficit (mEq/L)				
Parameters	Unstandardized B Coefficients	95% Confidence interval		*P*-value
		Lower bound	Upper bound	
Maternal age	0.052	−0.010	0.115	0.10
History of diabetes mellitus	0.709	−0.338	1.756	0.18
History of hypertension	0.185	−0.763	1.132	0.70
History of Preeclampsia	−0.244	−1.743	1.254	0.75
Delivery type	1.970	1.001	2.939	<0.001
Gravidity	0.090	−0.210	0.391	0.55
Parity	−1.188	−1.635	−0.740	<0.001
Gestational week	0.016	−0.139	0.171	0.84
UC – mean	−0.001	−0.026	0.025	0.96
UC – CV	−0.001	−0.011	0.010	0.90
FHR – mean	0.043	0.019	0.067	<0.001
FHR – CV	0.405	0.334	0.476	<0.001
Newborn sex	0.089	−0.439	0.617	0.74
Newborn weight (g)	0.000	0.000	0.001	0.54

Each 10-beat/min increase in the FHR was associated with a 0.01 decrease in the pH and a 0.43 mEq/L increase in the base deficit.

Each 10% increase in the CV of the FHR was associated with a 0.13 decrease in the pH and a 4.05 mEq/L increase in the base deficit.

### Association Between FHR and Apgar Scores

In multivariable adjusted models, the following factors were associated with an increased risk of low Apgar scores at birth: increased mean and CV of the FHR, history of preeclampsia, type of delivery (Caesarian section over vaginal), decreased parity (Tables 4, 5). The following parameters were not associated with risk of low Apgar scores at birth: maternal age, history of diabetes mellitus, hypertension, gravidity, uterine contractions, newborn sex or weight.

**Table 4:** Association between maternal and newborn parameters and Apgar 1 score (1 min after birth).

Apgar 1 score				
Parameters	Unstandardized B Coefficients	95% Confidence interval		*P*-value
		Lower bound	Upper bound	
Maternal age	−0.001	−0.031	0.029	0.94
History of diabetes mellitus	0.279	−0.247	0.805	0.30
History of hypertension	−0.279	−0.755	0.198	0.25
History of Preeclampsia	−0.767	−1.521	−0.014	0.05
Delivery type	−0.207	−0.678	0.264	0.39
Gravidity	−0.031	−0.181	0.120	0.69
Parity	0.367	0.144	0.589	0.001
Gestational week	0.165	0.087	0.242	<0.001
UC – mean	0.000	−0.013	0.012	0.95
UC – CV	−0.002	−0.007	0.003	0.44
FHR – mean	−0.020	−0.031	−0.008	0.001
, FHR – CV	−0.131	−0.166	−0.095	<0.001
Newborn sex	0.208	−0.056	0.471	0.12
Newborn weight (g)	−0.001	−0.001	0.000	<0.001

**Table 5:** Association between maternal and newborn parameters and Apgar 5 score (5 min after birth).

Apgar 5 score				
Parameters	Unstandardized B Coefficients	95% Confidence interval		*P*-value
		Lower bound	Upper bound	
Maternal age	−0.007	−0.028	0.015	0.54
History of diabetes mellitus	0.001	−0.374	0.376	0.99
History of hypertension	−0.126	−0.465	0.214	0.47
History of Preeclampsia	−0.938	−1.475	−0.400	0.001
Delivery type	−0.083	−0.419	0.253	0.63
Gravidity	−0.070	−0.177	0.038	0.20
Parity	0.234	0.076	0.393	0.004
Gestational week	0.053	−0.002	0.109	0.06
UC – mean	0.002	−0.007	0.011	0.63
UC – CV	0.000	−0.004	0.003	0.89
FHR – mean	−0.014	−0.023	−0.006	0.001
FHR – CV	−0.086	−0.111	−0.061	<0.001
Newborn sex	0.060	−0.128	0.248	0.53
Newborn weight (g)	0.000	−0.001	0.000	0.003

Each 10-beat/min increase in the FHR was associated with a 0.2 and 0.14 decrease in the Apgar score at 1 and 5 min after birth, respectively.

Each 10% increase in the CV of the FHR was associated with a 1.31 and 0.86 decrease in the Apgar score at 1 and 5 min after birth, respectively.

## Discussion

The main findings of this study are that in a cohort of uncomplicated childbirths without severe cases of neonatal morbidity, hypoxic ischemic encephalopathy, or seizures: (1) increased mean and CV of the intrapartum FHR were associated with increased risk of metabolic acidosis and low Apgar scores at birth; (2) FHR was not associated with maternal age, history of diabetes mellitus or hypertension or preeclampsia, delivery type, gravidity, parity, gestational week, mean of uterine contractions. Besides the intrapartum cardiotocography FHR, delivery type, and decreased parity were also associated with neonatal acidemia and the physical condition of a newborn infant.

Intrapartum fetal hypoxia represents an important cause of postnatal cerebral palsy or other neurologic outcomes and in a significant proportion of cases there is evidence of suboptimal care related to fetal surveillance. Umbilical artery metabolic acidosis is commonly used to detect neurological injury (Ross and Gala, 2002).

A three-tiered FHR interpretation system for intrapartum cardiotocography FHR tracing interpretation was proposed (Macones et al., 2008; American College of Obestetricians and Gynecologists, 2009). As our study did not include complicated pregnancies, it supports using the normal cardiotocography FHR (category I), which has a predictive value of 99.7% of an Apgar score more than 7 (Macones et al., 2008; Crovetto et al., 2017; Raghuraman and Cahill, 2017).

Using multivariable models to control confounders, cardiotocography FHR was recently found to be an independent predictor of fetal acidosis (Silberstein et al., 2017), respiratory morbidity in term neonates (Liu et al., 2015), and indicator for preterm cesarean delivery for increased risk of neonatal and childhood morbidity (Mendez-Figueroa et al., 2015). In our multivariable model analysis, metabolic acidosis at birth had an independent relationship with the cardiotocography FHR mean and variability and also with the type of delivery (Caesarian section over vaginal) and parity. Our results are in agreement with the study of Heinonen et al. who also found that parity, but not maternal age, was an independent risk factor for neonatal acidosis (Heinonen and Saarikoski, 2001). Corroborating our results with another study of women with a singleton term pregnancy that found previous cesarean delivery and nulliparity as risk factors for neonatal metabolic acidosis (Westerhuis et al., 2012) may indicate that not only previous but also the current cesarean delivery may represent an actual challenge to the fetus. In two studies of poor neonatal adaptation at birth with severe neonatal acidosis (umbilical artery pH less than 7.10) independent risk factors included abnormal cardiotocography FHR, maternal age 35 years or older, parity, prior neonatal death or cesarean delivery (Maisonneuve et al., 2011; Crovetto et al., 2017). Our data extends a valuable role for cardiotocography FHR in predicting neonatal acidosis in deliveries with Apgar 5 ranging from fairly low to normal without neurological complications.

Heart rate variability (HRV) analysis with search for new algorithms is commonly employed to measure alterations in autonomic tone with predictive value in diseases (Campos et al., 2013). We have identified the CV of the heart rate as a sensitive measure of autonomic dysfunction (Miyabara et al., 2017) and independently associated with vascular atherosclerosis (Pereira et al., 2017). In this study, we found that CV of intrapartum cardiotocography FHR is an independent predictor of neonatal acidemia and Apgar scores.

### Limitations and Strengths of the Study

There are important limitations associated with this study. First, since this was a retrospective study, we could only investigate factors that were available to us in the current dataset, mid-to-long term outcomes of the infants being not recorded in the database. Also, fetal sleep state was not considered in this study. Second, although robust statistical methods were used to account for differences between groups, the potential for residual confounding cannot be ruled out. This study, as with most retrospective studies, is susceptible to bias. Third, the results of the study cannot be generalizable to all patients, for instance to those with an APGAR score critically low or associated with neurological complications and did not include data from neonates with severe cases of neonatal morbidity, hypoxic ischemic encephalopathy, or seizures. Limitations notwithstanding there are key strengths to this paper. To our knowledge, this is the first study with a relatively large sample size providing sufficient statistical power to explore the relationship between intrapartum FHRV and neonatal acidemia or APGAR score. Also, we adjusted for several confounders including important comorbidities.

## Conclusion

In conclusion, the present findings provide evidence that intrapartum cardiotocography FHR could be a predictor for neonatal acidemia and the physical condition of a newborn infant, as determined by arterial cord blood pH and base deficit, Apgar 1, and 5 scores. Further studies are warranted to examine the potential relationship between intrapartum FHR and neonatal pathologies including neurological complications.

## Author Contributions

OB, LC, AB, MD, TM, and DdS conceived and designed the study, and analyzed and interpreted the data. OB, LC, MD, and AB drafted the manuscript. TM, MD, DdS, AB, LC, and OB critically revised the manuscript. LC and OB are the guarantors of this work and, as such, had full access to all the data in the study and takes responsibility for the integrity of the data and the accuracy of the data analysis.

## Conflict of Interest Statement

The authors declare that the research was conducted in the absence of any commercial or financial relationships that could be construed as a potential conflict of interest.

## Footnotes

1 https://physionet.org/physiobank/database/ctu-uhb-ctgdb/
